# The influence of tart cherries (*Prunus Cerasus*) on vascular function and the urinary metabolome: a randomised placebo-controlled pilot study

**DOI:** 10.1017/jns.2021.68

**Published:** 2021-09-13

**Authors:** Rachel Kimble, Lucy Murray, Karen M. Keane, Karen Haggerty, Glyn Howatson, John K. Lodge

**Affiliations:** 1 Department of Sport, Exercise and Rehabilitation, Faculty of Health and Life Sciences, Northumbria University, Newcastle-upon-Tyne, UK; 2 Department of Applied Sciences, Faculty of Health and Life Sciences, Northumbria University, Newcastle-upon-Tyne, UK

**Keywords:** Anthocyanin, Arterial stiffness, Blood pressure, Cherry, Metabolomics

## Abstract

Montmorency tart cherries (MC) have been found to modulate indices of vascular function with interventions of varying duration. The objective of this preliminary study was to identify the chronic effects of MC supplementation on vascular function and the potential for urinary metabolomics to provide mechanistic evidence. We performed a placebo-controlled, double-blind, randomised study on 23 healthy individuals (18M, 7F) that consumed 30 ml MC or a placebo twice daily for 28 days. Whole body measures of vascular function and spot urine collections were taken at baseline and after supplementation. There were no significant changes to vascular function including blood pressure and arterial stiffness. Urinary metabolite profiling highlighted significant changes (*P* < 0⋅001) with putative discriminatory metabolites related to tryptophan and histidine metabolism. Overall, MC supplementation for 28 days does not improve indices of vascular function but changes to the urinary metabolome could be suggestive of potential mechanisms.

## Introduction

Cardiovascular disease (CVD) is the primary cause of morbidity and mortality^(1)^. Preclinical studies suggest that Montmorency tart cherries (MC) can positively impact major risk factors of CVD, activities thought to be related to their phytochemical content^(2)^. However, data from human trials are less clear. MC have been shown to reduce systolic blood pressure (SBP) after acute^(3,4)^, short-term^(5)^ and chronic^(6)^ intake and to improve vascular dysfunction^(7)^, reduce LDL-cholesterol^(6)^ and oxidised LDL^(8)^, presumably due to their antioxidant and anti-inflammatory activities^(2)^. Notwithstanding, other studies have not shown the benefits of MC on SBP or vascular function^(8–10)^. Studies are difficult to interpret with different populations, dosing and durations but we have previously demonstrated that a dosing regimen of 30 ml twice daily (equivalent to 60 ml/d) significantly affected vascular function in mildly hypertensive males^(3)^ and middle-aged adults^(4)^. There is also a lack of understanding for any underlying mechanisms, and to provide mechanistic evidence, the use of metabolomics has become increasingly popular. For example, metabolomics have shown that anthocyanin-rich interventions increase circulating polyphenol metabolites that might account for improvements in vascular function^(11)^. Therefore, the aim of this preliminary study was to characterise the effects of MC supplementation with an effective dosage regimen on vascular function with a 4-week intervention and explore if metabolomics can provide mechanistic evidence.

## Methods

A power calculation was performed for the primary outcome systolic blood pressure. According to the previous study^(3)^ and using a population standard deviation of 11 mm Hg at 80 % power and 5 % significance, the minimum number of participants required to allow detection of a difference of 5 mm Hg (clinically relevant outcome) between the responses to the two intervention drinks was estimated to be 12. Twenty-three healthy non-smoking volunteers, who did not regularly consume cherries, use antioxidant supplements or medications, took part in the present randomised (http://randomisation.eu/index.shtml), double-blind, placebo-controlled, parallel study. Participants attended the laboratory on two separate occasions. Participants’ vascular function measures and spot urine samples^(12)^ were collected following an overnight fast (≥12 h) pre and post 4-week supplementation with either MC concentrate (m/f: 9/3) or an isocaloric placebo (m/f: 8/3). Baseline characteristics are shown in Table 1 and there was a significant difference in age (*P* = 0⋅03). Participants maintained their habitual diet apart from following a low phenolic diet for 48 h before each laboratory visit. Participants consumed 30 ml of an MC concentrate (CherryActive, UK; containing 36⋅8 mg of anthocyanins (as cyanidin-3 glucoside equivalents using the pH differential colorimetric method) as well as several hydroxycinnamic and hydroxybenzoic acids) diluted in 100 ml of water or the same amount of placebo twice daily, once in the morning and evening. The placebo was a low fruit (<1 %) unsweetened black cherry cordial mixed with ingredients to match MC concentrate for energy and macronutrients (Per 130 ml, energy 102 kcal, carbohydrate 24⋅5 g, protein 1⋅1 g and fat 0 g), colour and taste as described^(3,4)^. No adverse effects were reported and average self-reported compliance was 86 %. This study was conducted according to the guidelines laid down in the Declaration of Helsinki and all procedures were ratified by Northumbria University's Research Ethics Committee, and participants provided written informed consent. The trial conforms to CONSORT standards and is registered with ClinicalTrials.gov, identifier NCT04840160.

**Table 1.** Baseline characteristics of participants

	Tart cherry group (*n* 12)	Placebo group (*n* 11)	*P-*value
Sex (m/f, *n*)	9/3	8/3	–
Age (years)	24⋅7 ± 3⋅6	22⋅0 ± 2	0⋅03
BMI (kg/m^2^)	24⋅6 ± 3⋅2	24⋅6 ± 2⋅3	0⋅98
SBP	124⋅1 ± 10⋅7	119⋅6 ± 10⋅7	0⋅32
DBP	67⋅6 ± 5⋅6	65⋅1 ± 6⋅7	0⋅11

Vascular function was assessed as we have detailed elsewhere^(13)^ using appropriate commercial devices for blood pressure and heart rate (HR) (M10-IT; Omron Healthcare), pulse wave velocity (PWV) (SphygmoCor CPV system ScanMed Medical, UK) and Digital Volume Pulse (DVP) (PulseTrace PCA 2 with a photoplethysmograph transducer, MicroMedical, UK). Urine samples were prepared for metabolomic analysis as described^(12)^ following standardisation by refractive index. Hydrophilic interaction liquid chromatography (HILIC) analysis was deemed more appropriate for a more polar biological fluid such as urine. HILIC was conducted using ultrahigh resolution liquid chromatography (UHPLC) and mass spectrometry (MS) (adapted from Langer et al.^(12)^) and data acquisition parameters, data processing and data mining information are detailed in the online supplementary methods. Statistical analysis was conducted using SPSS for windows (V24.0: Chicago, IL). A two-way repeated-measures ANOVA analysed the effect of time, treatment and time × treatment interactions for vascular function measures. Baseline characteristics were compared between groups by an unpaired *t* test. The *α* level for statistical significance was set at 0⋅05 *a priori*.

## Results and discussion

Despite accumulating evidence for the potential role of cherries in cardiovascular health^(2)^, the exact mechanisms have yet to be fully elucidated. Thus, we incorporated a metabolomic approach to explore potential mechanisms. We found MC concentrate to have no significant effect on our indices of vascular function (SBP, diastolic blood pressure, MAP, HR, PWV, DVP-SI or DVP-RI) compared to the placebo after the intervention (*P* > 0⋅05; Table 2) with only minor changes from baseline, in line with evidence that MC has no effect on vascular function in normotensive individuals^(8–10,14)^, even those with higher doses^(15)^ or longer duration^(14,15)^. MC concentrate has been repeatedly shown to result in transient reductions in SBP that return to baseline within 3–4 h^(3)^. Since this study investigated vascular function after an overnight fast it is possible that any changes in BP were missed due to the seemingly rapid absorption and/or excretion of tart cherry anthocyanin metabolites^(16)^. It is likely that a combination of dose, duration and population are responsible for the null effects. Durations between 20 days^(5)^ to 12 weeks^(6)^ have been reported with no association between duration and effect. There is a better likelihood of finding a change in a higher risk population, as in a recent review of factors influencing the effects of anthocyanins, both a higher initial BP and habitual flavonoid intake were identified as relevant determinants of efficacy^(17)^, and these are points to inform future studies with MC concentrate.

**Table 2.** Influence of Montmorency cherry concentrate on vascular function

	Montmorency cherry (*n* 12)	Change from mean (% change)	Placebo (*n* 11)	Change from mean (% change)	*P*-value for ANOVA		
					Treatment	Time	Interaction
SBP (mmHg)							
Baseline	124⋅1 ± 10⋅7	−3⋅57 ± 10⋅2 (0⋅03%)	119⋅6 ± 10⋅7	1⋅82 ± 3⋅5 (−0⋅03 %)	0⋅21	0⋅18	0⋅84
4 weeks	127⋅5 ± 16⋅4		121⋅8 ± 11⋅1				
DBP (mmHg)							
Baseline	67⋅6 ± 5⋅6	−0⋅83 ± 7⋅1 (0⋅01%)	65⋅1 ± 6⋅7	−2⋅18 ± 12⋅8 (0⋅02%)	0⋅28	0⋅12	0⋅58
4 weeks	66⋅8 ± 6⋅3		63⋅3 ± 7⋅9				
MAP (mmHg)							
Baseline	86⋅5 ± 4⋅4	−1⋅7 ± 5⋅9 (0⋅02%)	83⋅3 ± 6⋅9	0⋅5 ± 6⋅2 (0⋅01%)	0⋅07	0⋅97	0⋅67
4 weeks	87⋅0 ± 7⋅5		82⋅8 ± 7⋅7				
HR (BPM)							
Baseline	66⋅7 ± 14⋅1	−4⋅33 ± 12⋅9 (0⋅09%)	66⋅5 ± 10⋅1	−1⋅5 ± 6⋅1 (0⋅03%)	0⋅71	0⋅25	0⋅75
4 weeks	69⋅8 ± 11⋅2		68⋅0 ± 9⋅2				
PWV (m/s)							
Baseline	5⋅5 ± 0⋅7	−0⋅22 ± 0⋅94 (0⋅05%)	5⋅7 ± 1⋅3	0⋅96 ± 1⋅82 (−0⋅17%)	0⋅72	0⋅36	0⋅2
4 weeks	6⋅1 ± 1⋅2		5⋅6 ± 0⋅8				
DVP-SI (m/s)							
Baseline	6⋅0 ± 0⋅8	−0⋅02 ± 0⋅51 (0⋅17%)	5⋅6 ± 0⋅7	0⋅23 ± 0⋅51 (0⋅17%)	0⋅19	0⋅45	0⋅24
4 weeks	6⋅0 ± 0⋅9		5⋅4 ± 0⋅5				
DVP-RI (%)							
Baseline	62⋅4 ± 13⋅4	−2⋅27 ± 16⋅3 (0⋅02%)	51⋅4 ± 10⋅6	−5⋅4 ± 9⋅8 (0⋅02%)	0⋅12	0⋅22	0⋅56
4 weeks	63⋅5 ± 15⋅9		56⋅8 ± 8⋅6				

Values are mean ± sd for SBP, DBP, MAP, HR, PWV, DVP-SI, DVP-RI.

SBP, systolic blood pressure; DBP, diastolic blood pressure; MAP, mean arterial pressure; HR, heart rate; PWV, pulse wave velocity; DVP-SI, digital volume pulse stiffness index; DVP-RI, digital volume pulse reflection index.

**Fig. 1.**
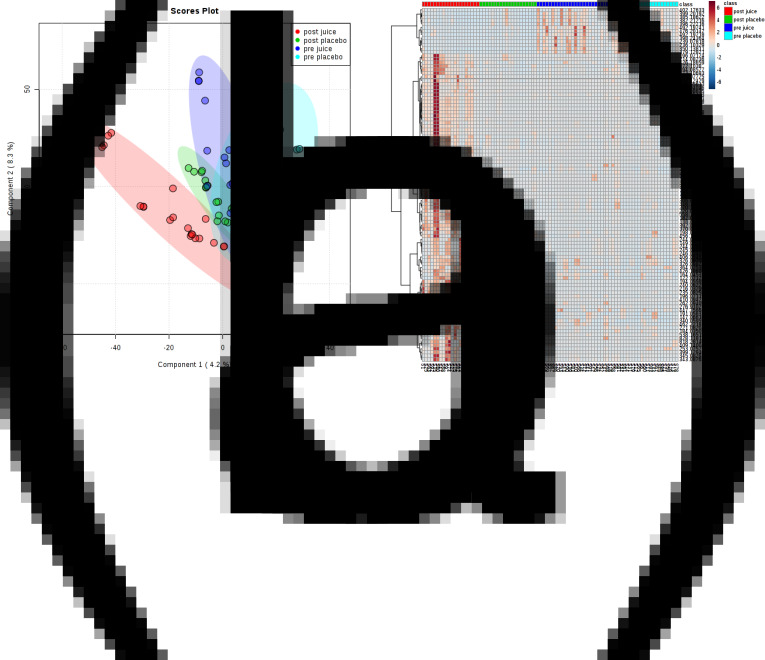
Multivariate analysis of the effect of cherry juice treatment on the urinary metabolome – (a) shows a score plot of a 4 component partial least squares-discriminant analysis model of all treatments, whilst a heat map of the same data is shown in (b). Negative ion mode data are shown as an example.

However, we did observe an influence on the urinary metabolome (Fig. 1). There was a good similarity between the metabolome of both treatments at baseline despite limited dietary restrictions and we were able to discriminate post-treatment MC concentrate samples (Fig. 1). Preliminary analysis of the top-ranked discriminatory features found 32 and 18 ion clusters in positive and negative mode, respectively, and Table 3 shows putative matches from these. These included metabolites from tryptophan metabolism (enrichment factor 7⋅1, −log10(p) 1⋅5) including 6-hydroxy melatonin, 5-hydroxyindoleacetate and melatonin in positive mode, while histidine metabolism was prominent in negative ion mode (enrichment factor 15⋅2, −log10(p) 1⋅1) suggesting an influence on tryptophan and histidine pathways even in the absence of changes to vascular function variables. For example, we observed a decrease in urinary excretion of melatonin and 6-hydroxymelatonin. Both tryptophan and melatonin are found in tart cherries^(2)^ and have been linked to improvements in sleep following MC supplementation as we have previously shown^(18)^. Moreover, melatonin and tryptophan metabolites might be beneficial to cardiovascular health because of their antioxidant, anti-inflammatory and antiatherogenic actions^(19)^, suggesting the properties of the indolamine compounds in MC warrant further attention. We also identified an increase in metabolites from the histidine pathway (e.g. histidine, methylated histidine), supporting a study supplementing anthocyanin-rich red wine that resulted in increases in urinary 1-methylhistidine^(20)^. We also found matches to expected exogenous metabolites, such as cyanidin glucosides, ferulic acid; however, these were not highly discriminatory, perhaps as they are rapidly cleared^(16)^. Although only preliminary findings, these data provide evidence that intakes of anthocyanin-rich beverages such as MC juice can influence amino acid metabolism and tentatively provide a mechanistic basis for their health effects, although in the present study these changes were not sufficient to elicit a physiological effect.

**Table 3.** Putative identifications of highly ranked discriminating metabolites following tart cherry juice supplementation

Annotation	Predicted formula	VIP	*m/z*	Adduct	Mass error (PPM)	Ion mode	Change	ID
*N*-Methyltryptamine	C11H14N2	3⋅38	207⋅14912	M + CH_3_OH + H	0	+	↓	HMDB0004370
		3⋅26	207⋅14904	M + CH_3_OH + H	1	+	↓	
		3⋅07	235⋅18057	M + IsoProp + H	2	+	↓	
5-Methoxydimethyltryptamine	C13H18N2O	2⋅65	251⋅17564	M + CH_3_OH + H	1	+	↓	HMDB0002004
5-Methoxytryptamine	C11H14N2O			M + IsoProp + H	1	+	↓	HMDB0004095
Nb-Acetyl-Nb-methyltryptamine	C13H16N2O	3⋅65	249⋅16001	M + CH_3_OH + H	1	+	↓	HMDB0029837
		3⋅43	249⋅16017	M + CH_3_OH + H	2	+	↓	
Melatonin	C13H16N2O2	2⋅66	233⋅16493	M + H	0	+	↓	HMDB0001389
		1⋅10	296⋅13704	M + ACN + Na	0	+	↓	
6-Hydroxymelatonin	C13H16N2O3	1⋅17	271⋅10589	M + Na	2	+	↓	HMDB0004081
		1⋅05	249⋅12377	M + H	2	+	↓	
		3⋅76	205⋅13356	M − CO_2_ + H	1	+	↓	
5-Hydroxyindoleacetate	C10H11NO2	4⋅24	194⋅05986	M + H	1	+	↑	HMDB0000763
1-Methylhistidine	C5H6N2O	3⋅8469	150⋅06697	M − H_2_O − H	1	−	↑	HMDB0000001
l-histidine	C6N9N3O2	4⋅2199	136⋅05121	M − H_2_O − H	1	−	↑	HMDB0000177
*N*,*N*-dimethylhistidine	C6H8N2O	3⋅1115	164⋅08275	[M − H_2_O − H]-	1	−	↓	HMDB0033438

VIP, variable importance in projection.

This study has limitations. We investigated a young, normotensive population, but individuals with higher CVD risk might benefit more from anthocyanin supplementation, or treatment with a longer duration and a higher dosing regimen and a larger number of participants. Notably, the population in the present study was not confounded by medications. Secondly, our dosing regimen was 4-week long and this duration may need to be extended, but importantly the intervention was well tolerated. Furthermore, a crossover study may be more optimal (and especially for metabolomics) as this would reduce inter-individual variation. This study suggests that consumption of MC concentrate for 4 weeks does not influence indices of vascular function in healthy individuals. However, the intervention did induce changes to the urinary metabolome specific to amino acid metabolism and this is the first such report of this effect. These findings have important implications for the physiological effects of tart cherries. Future studies, with larger sample sizes, with higher risk populations and of longer duration are needed to fully elucidate the potential of this intervention but do suggest that metabolomics can be useful to identify metabolic changes indicative of mechanisms of action.

## Supplementary material

For supplementary material accompanying this paper visit http://dx.doi.org/10.1017/S2048679021000689. click here to view supplementary material
