# Supplementary material for: The influence of tart cherries (Prunus Cerasus) on vascular function and the urinary metabolome: a randomised placebo-controlled pilot study
Source: J Nutr Sci. 2021 Sep 13;10:e73. doi: 10.1017/jns.2021.68 (PMC8453453; doi:10.1017/jns.2021.68)
Supplement: Supplementary file 1 [file jnssup.zip › S2048679021000689sup002.docx]

**Supplementary Methods**

**Study design**

The present study employed a randomised, double-blind, placebo-controlled, parallel design. Once baseline variables were collected, participants were randomly assigned to receive either Montmorency tart cherry (MC) concentrate or an isocaloric placebo (PLA). Participants were instructed to consume their intervention treatment for 4 weeks, after which outcome variables were reassessed under identical testing conditions. Primary endpoints were systolic and diastolic blood pressure, while secondary endpoints were pulse wave velocity, digital volume pulse, and untargeted urinary metabolomics.

**Subjects**

Exclusion criteria were hypertension, cardiometabolic diseases, regular consumption of cherries, use of any antioxidant supplements or medications. Participants were in good apparent health as assessed by a general health questionnaire. Recruitment was via general emails to staff and post-graduate students of Northumbria University and poster advertisements across the University campus. In total twenty-three healthy participants completed this study, two additional participants were baseline tested however were unable to attend the post intervention testing; their baseline measures were not included in any of the data analysis.

**Urinary metabolomic analysis**

Hydrophilic interaction liquid chromatography (HILIC) based analysis of urine samples was conducted using ultrahigh resolution liquid chromatography (UHPLC) and mass spectrometry (MS). Metabolite profiles of urine samples were generated on a Dionex 3000 Ultra High Pressure Liquid chromatography (UHPLC) system hyphenated to the Q-Exactive classic high resolution mass spectrometer system (ThermoScientific, Bremen, Germany). All solvents and ionization agents used were of analytical grade or higher unless stated. The chromatographic separation was performed on a Water Acquity Ethylene Bridge Hybrid (BEH) Amide analytical column (2.1 x 150mm) with particle size of 1.7micron at a flow rate of 400µL/min, the column temperature was set to 45^o^C. The Binary buffer system was as follows: Buffer A was MilliQ water and Buffer B was acetonitrile, both with 10mM ammonium formate adjusted to pH 3.5 using formic acid. The LC profile was as follows: T:0 min: 90%(B), T: 2min 60% (B) T: 5min 40%(B), T:7.5 min 40%(B), T:7.6min 90%(B), T:10 min 90% (B). A 3µL injection was applied. The Heated spray ionization source (HESI) was set with a Sheath gas flow rate of 50 and Aux gas flow rate of 13. The Spray voltage was set to 3.5kV with a Capillary temperature of 275^o^C. The Aux gas heater temperature was adjusted to 425^o^C. The mass (MS1) acquisition range was 75-1000 m/z units at a mass resolution of 35,000 with 7.6 scans per second. The data was acquired on both Positive and Negative mode polarity (independently), the setting for the negative mode was the same as positive ion mode except the voltage was set to 2.5kV. The system was primed with a minimum of 10 sequential injections of pooled QC to stabilise the HESI source and to check for chromatographic stability before initialling the batch analysis. Peak intensity table generation and alignment were performed using compound discoverer 2.1 (ThermoScientific, Bremen, Germany) with an alignment window of 0.25min, mass tolerance: 5ppm and a signal intensity threshold of 200,000 counts with a signal to noise ratio of 5:1.

**Characterisation and identification of discriminating features**

Peak intensity tables from Compound Discoverer were processed using MetaboAnalyst© software ^(^[^1^](#_ENREF_1)^)^. The full dataset was autoscaled and log transformed. MetaboAnalyst performed detailed multivariate and univariate analysis including Principal Component Analysis that was used for data quality control and Partial Least Squares Discriminant Analysis (PLSDA) that was used to test for discrimination between sample groups. Cross validation tests were used to test the robustness of the model, using Q2 and R2 and classification metrics, while Variable Importance in Projection (VIP) data was used to rank the most discriminatory species. We established both 4 component (all treatments) and 2 component (pre versus post) PLSDA models, the latter were used to rank discriminatory species. Only those features not significantly changed in placebo treatments were taken forward for identification. A number of approaches were used to putatively identify metabolites in the absence of fragmentation data. Firstly, the top discriminating features (VIP > 3) underwent pattern searching (Pearson’s correlation) and features with similar behaviour were identified as ion clusters that could originate from the same compound as different adducts. Then putative identification of these features was performed using established databases (e.g METLIN, the Human metabolome database) to identify adducts of the same compound. Secondly, polyphenol specific databases PhytoHub, PhenolExplorer and the USDA flavonoid database were searched for expected polyphenol compounds found in tart cherries and their relevant metabolites for expected m/z values in each ionisation mode. Lastly, to support putative identifications, ions that were significantly different in comparison of means testing underwent metabolic pathway enrichment analysis using the mummichog algorithm ^(^[^2^](#_ENREF_2)^)^ that uses the p value and t-score to map all possible metabolite matches to a network to determine enrichment. A mass accuracy of 5 ppm was used and the KEGG and MTF platforms used for enrichment searches. Annotation of identified metabolites was carried out according to level 2 of identification proposed by the Metabolomics Standards Initiative ^(^[^3^](#_ENREF_3)^)^.

1. Xia J, Wishart DS (2016) Using MetaboAnalyst 3.0 for comprehensive metabolomics data analysis. *Current protocols in bioinformatics* 55, 14.10. 11-14.10. 91.

2. Li S, Park Y, Duraisingham S *et al.* (2013) Predicting network activity from high throughput metabolomics. *PLoS Comp Biol* 9, e1003123.

3. Sumner LW, Amberg A, Barrett D *et al.* (2007) Proposed minimum reporting standards for chemical analysis. *Metabolomics* 3, 211-221.
