# Supplementary material for: The influence of tart cherries (Prunus Cerasus) on vascular function and the urinary metabolome: a randomised placebo-controlled pilot study
Source: J Nutr Sci. 2021 Sep 13;10:e73. doi: 10.1017/jns.2021.68 (PMC8453453; doi:10.1017/jns.2021.68)
Supplement: Supplementary file 1 [file jnssup.zip › S2048679021000689sup001.doc]

**CONSORT 2010 Flow Diagram**

**Allocation**

**Analysis**

**Follow-Up**

**Enrollment**

Assessed for eligibility (n=25)

Excluded (n=0)

♦  Not meeting inclusion criteria (n=0)

♦  Declined to participate (n= 0)

♦  Other reasons (n=0)

Analysed (n=12)
♦ Excluded from analysis (n=0)

Lost to follow-up (give reasons) (n=0)

Discontinued intervention (give reasons) (n=0)

Allocated to MC concentrate intervention (n=12)

♦ Received allocated intervention (n=12)

♦ Did not receive allocated intervention (n= 0)

Lost to follow-up (give reasons) (n=0)

Discontinued intervention (give reasons) (n=0)

Allocated to placebo intervention (n=13)

♦ Received allocated intervention (n=11)

♦ Did not receive allocated intervention (personal reasons) (n=2)

Analysed (n=11)
♦ Excluded from analysis (n=0)

Randomized (n=25)
